# Education rather than age structure brings demographic dividend

**DOI:** 10.1073/pnas.1820362116

**Published:** 2019-06-10

**Authors:** Wolfgang Lutz, Jesus Crespo Cuaresma, Endale Kebede, Alexia Prskawetz, Warren C. Sanderson, Erich Striessnig

**Affiliations:** ^a^Wittgenstein Centre for Demography and Global Human Capital (International Institute for Applied Systems Analysis, Vienna Institute of Demography/Austrian Academy of Sciences, Vienna University of Economics and Business Administration), International Institute for Applied Systems Analysis, Laxenburg, Lower Austria 2361, Austria;; ^b^Department of Economics, Vienna University of Economics and Business, 1020 Vienna, Austria;; ^c^Research Unit Economics, Institute of Statistics and Mathematical Methods in Economics, TU Wien, 1040 Vienna, Austria

**Keywords:** demography, economic growth, education, age structure

## Abstract

The relationship between population changes and economic growth has been debated since Malthus. Initially focusing on population growth, the notion of demographic dividend has shifted the attention to changes in age structures with an assumed window of opportunity that opens when falling birth rates lead to a relatively higher proportion of the working-age population. This has become the dominant paradigm in the field of population and development, and an advocacy tool for highlighting the benefits of family planning and fertility decline. While this view acknowledges that the dividend can only be realized if associated with investments in human capital, its causal trigger is still seen in exogenous fertility decline. In contrast, unified growth theory has established human capital as a trigger of both demographic transition and economic growth. We assess the relative importance of changing age structure and increasing human capital for economic growth for a panel of 165 countries during the time period of 1980–2015. The results show a clear dominance of improving education over age structure and give evidence that the demographic dividend is driven by human capital. Declining youth dependency ratios even show negative impacts on income growth when combined with low education. Based on a multidimensional understanding of demography that considers education in addition to age, and with a view to the additional effects of education on health and general resilience, we conclude that the true demographic dividend is a human capital dividend. Global population policies should thus focus on strengthening the human resource base for sustainable development.

## Significance

Global environmental change and discussions about the drivers of international migration lead to renewed interest in population growth and global demographic change. The notion of the demographic dividend was introduced to highlight the benefits of fertility decline, yet, among African leaders, it is also often interpreted as describing the benefits of their youthful populations. Due to its controversial nature, the topic of population was not explicitly included in the Sustainable Development Goals. In this controversial discussion, this paper provides a systematic reassessment about what aspects of demographic change have beneficial consequences for economic growth and sustainable development.

The notion of a demographic dividend has recently received prominence in the discussions around international development as a particular way of viewing the effects of demographic changes on economic growth. The original concept is based on the assumption that a decline in the proportion of young people, as a consequence of reduced fertility in a high-fertility context, will give a boost to economic growth if investments in education and health services as well as economic policies conducive to income growth are implemented. While international agencies and foundations promoting family planning tend to emphasize the role of low fertility (1), policy makers in Africa tend to highlight the advantage of the human capital associated with a youthful population (2).

This contradictory use of the term “demographic dividend” further adds to an already complex discussion about the effects of demographic trends on economic growth in the international development community. The topic has been discussed at least since Thomas Malthus published his 1798 essay on “The Principle of Population” (3). The focus of the discussion on the role of population change in economic consequences has moved from an early focus on population growth to a focus on changing population age structures (since the 1980s) to a focus on changing age and educational attainment structures (since the 2000s). Here we revisit the discussions in the demographic and economic research communities and provide empirical estimates of such a possible demographic dividend on the basis of a multidimensional demographic approach applied to a panel of over 165 countries for the period 1980–2015.

## Population and Economic Development

In reaction to a highly controversial debate over population growth that ranged from horror about a “population bomb” (4) to praising more people as the “ultimate resource” (5), the National Research Council (NRC), through its authoritative 1986 report (6), assessed the global empirical evidence related to the possible benefits of lower population growth, ranging from less degradation of natural resources to effects on savings, innovation, and per capita expenditure on schooling and health. It prominently highlighted the importance of human capital for economic growth and offered a differentiated overall assessment of the effects of population growth by pointing at important conditionalities. Our reassessment of the evidence 33 y after this report confirms the importance of conditionality and, based on newly available detailed data on educational attainment distributions by age and sex, we can single out human capital formation as a key strategy among a host of relevant government policies.

This differentiated conclusion of the NRC report came as a disappointment to the proponents of family planning, who had hoped for clearer evidence on the economic benefits of fertility decline by itself. Such evidence seemed to regain importance with a shifting of the focus from population growth to the changes in age structure that are induced by declining fertility. Such an approach builds on a tradition of the study of age structure effects which starts, to our knowledge, with the little-known work of Günther (7) for Germany in 1931, claiming that declining birth rates cause unemployment because of the associated declines in consumer demand. The more prominent work by Coale and Hoover (8) focusing on capital dilution through many children also had an implicit focus on age structure. The empirical work by Kelley and Schmidt (9, 10), Bloom and Williamson (11), and other related studies estimated cross-country growth regressions which explicitly included the ratio of the working-age population over total population as one of the determinants of economic growth. These studies tend to find significant effects of an increasing share of the working-age population on output per person, not only through the increasing proportion of potentially productive individuals (also labeled translation effect) but also through the productivity effect [measured by gross domestic product (GDP) per person of working age], which would presumably result from higher aggregate savings and more investments in infrastructure, as well as higher female labor force participation rates.

It is worth noting that “working age” is an abstraction—typically assuming that work only happens in the 15- to 65-y age range and that everybody makes an equal contribution—derived from Western welfare states and from a time when it was difficult to get actual data on labor force participation and educational attainment, by age and sex. With good national time series data on educational attainment, by age and sex, available, there is no reason to further uphold the highly problematic assumption that everybody of a certain age is equally contributing to economic growth.

Recently, a set of global economic growth regression studies has explicitly included human capital variables in addition to indicators of age structure. While Bloom and Williamson (11) do, in fact, include years of postprimary schooling of the adult population in their first set of regressions (with a positive and statistically significant effect on economic growth), they do not include it in their final model, which results in the widely cited finding that about a third of the economic growth of the Asian tigers can be explained by age structure changes, with the role of human capital not being discussed. Crespo Cuaresma and coworkers (12, 13) provide a global assessment of the relative effects of age structure and human capital using the specification by Benhabib and Spiegel (14, 15). They include the absolute level of education as a factor facilitating technological innovation and adoption (and thus affecting total factor productivity), in addition to the rate of change in the human capital of the labor force. The empirical results in Crespo Cuaresma et al. (13) suggest strong human capital effects on economic productivity but no significant productivity effect of changing age structures. Only a quantitatively small translation (accounting) effect of the age structure remains, resulting from the fact that GDP per person is sensitive to the number of children included in the denominator. These results let the authors conclude that the demographic dividend is in fact an education dividend.

Aside from the population research community, the importance of human capital for long-run economic growth has been demonstrated during recent years within the framework of the so-called Unified Growth Theory (16). While low technological progress in the Malthusian phase of history implied that population growth induced pressure on economic growth, increasing technological progress allowed for the continuous increase of population and economic growth in the post-Malthusian phase. The transition to the modern growth regime (on which our paper focuses) was then initiated by increasing human capital triggering the demographic transition that finally led to a negative relation between economic growth and the rate of population growth. On the supply side, this model assumes that increasing returns to human capital accumulation, as caused by technological progress (17), induce higher investment into the education of children and a reduction in fertility, thereby initiating the onset of the demographic transition. The mutual causation between education and fertility decline has been empirically verified (e.g., for Prussia in ref. 18, and for Ireland in ref. 19), and complementary theories (20) stress the role of increasing life expectancy (that boosted the returns to human capital further) and the importance of formal schooling in the period after the Industrial Revolution. The resulting positive feedback loop between technological progress, human capital formation, fertility decline, and increasing survival is the underlying mechanism that explains the modern growth regime.

So far, most of the studies on the connection between demographic change and economic growth have focused on the role of changes in age structure (as induced by the demographic transition), assigning the human capital component only a mediating role. However, as the vast literature in economics shows, human capital is the key driver to explain both the demographic transition and economic growth. Our results strongly support this theoretical literature, since we identify the compositional change of human capital as the main correlate to explain economic growth.

## Changing Age and Education Structures

While, sometimes, demographic change is narrowly viewed as only referring to changing age structure, both common usage by the public and authoritative scientific definitions have a broader view that includes changes with respect to several demographic dimensions. When media write about the changing demographics of America in the context of voting behavior, they refer to the changing proportions of Hispanics, changing proportions in urban, semiurban, and rural areas, and changing education structures, among others. This is also in line with the definition of demography by the International Union for the Scientific Study of Population (IUSSP) as the scientific study of changing population size and structures, addressing multiple structures.* The influential textbook *Methods and Materials of Demography* (21, 22) denotes as “demographic” to all characteristics of people that are typically collected in a census. Following this broader definition, we call changes in the education structure of populations demographic in the same way as changes in the age structure. Terminologically, a demographic dividend can thus also be a dividend arising from changing education structures. Which of these demographic structures is more important for economic growth is a matter of empirical assessment rather than *ex ante* assumptions.

Using tools of multidimensional population dynamics (23, 24), the changing structures of educational attainment by age and sex have recently been reconstructed for all countries in the world back to 1950 and projected to the end of the century according to different scenarios. Unlike other historical data on human capital (25), these human capital data are based on models that also take into account that vital rates differ by level of education (26–28). With most countries showing strong fertility and mortality differentials by education, explicitly incorporating these additional sources of population heterogeneity also tends to change the aggregate-level results (population size and age structure) in addition to providing useful information about these additional dimensions themselves (such as changing educational attainment distributions).

**Fig. 1.**
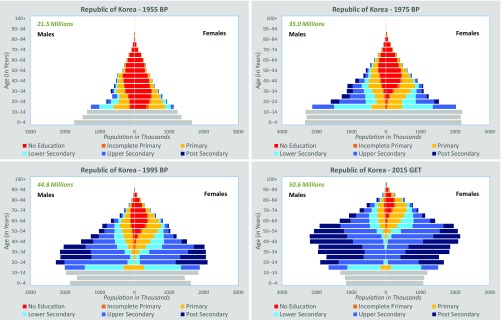
Republic of Korea age and education pyramids for 1955, 1975, 1995, and 2015.

Fig. 1 illustrates reconstructed education and age pyramids for South Korea, a country that has featured prominently in the discussions around the potential benefits of the demographic dividend. The figure clearly indicates that basic education expanded massively among young cohorts before economic growth took off with double-digit rates in the late 1960s. These cohort-specific educational attainment data also helped to unambiguously demonstrate the effect of human capital on economic growth, a link that had previously been blurred by the fact that statistical signal was lost when using the mean years of schooling of the entire adult population as an indicator of human capital rather than accounting for decisive differences by age cohorts (12).

## Assessing the Interaction between Age Structure and Education for Economic Growth

We adopt the theoretical framework put forward by Kelley and Schmidt (10) and expand it to account for technology adoption effects such as those proposed in Benhabib and Spiegel (14, 15) or Lutz et al. (12). The production function of the economy is assumed to be given by a Cobb−Douglas specification with Hicks-neutral technical change. Output is produced by physical capital that evolves based on the savings decisions of households and depreciates over time, and by human capital determined by the composition of the population by age, education, and labor force participation. Technological change, which increases the efficiency of all factors of production, is, in turn, affected by the level of human capital, as well as by the interaction between human capital and the country’s technological “backwardness” (the distance between the technology level of the country and that of the global frontier).

Such a model predicts effects of age structure which are determined by the prevailing level of human capital. In other words, the effects of a given change in age structure on economic growth depend on whether it takes place in a highly educated context or in the framework of a largely illiterate society. From a theoretical point of view, the effect of human capital on the speed of income convergence implies that relatively poorer economies benefit more from human capital accumulation than richer ones, due to technology adoption effects, which speed up the process of convergence to the technology frontier (14, 15). The effects of age structure (as captured by the share of working-age population) on economic growth depend on the stock of human capital, with higher human capital stocks leading to larger positive effects of increases in working-age population relative to the total population.

The regression model resulting from the theoretical specification used (*SI Appendix*) is given by

$$\Delta \ln\frac{Y_{it}}{N_{it}}=\varphi_{1}h_{it}+\varphi_{2}\ln\frac{Y_{it-1}}{N_{it-1}}+\varphi_{3}h_{it}\ln\frac{Y_{it-1}}{N_{it-1}}+\varphi_{4}\ln\frac{W_{it-1}}{N_{it-1}}+\varphi_{5}h_{it}\ln\frac{W_{it-1}}{N_{it-1}}+\varphi_{6}\ln\frac{L_{it-1}}{W_{it-1}}++\varphi_{7}h_{it}\ln\frac{L_{it-1}}{W_{it-1}}+\varphi_{8}\Delta \ln k_{it}+\varphi_{9}\Delta \ln L_{it}+\varphi_{10}\Delta \ln N_{it}+\varepsilon_{it},$$ [1]

where $Y_{it}$ is total output in country *i* at time *t*, $k_{it}$ denotes physical capital per worker, $h_{it}$ measures human capital, $N_{it}$ is total population, $W_{it}$ is working-age population, and $L_{it}$ is the size of the labor force. The error term, $\varepsilon_{it}$, is assumed to contain a fixed country-specific component and a period component, as well as an independent random component. We estimate the specification put forward above making use of a panel dataset for over 165 countries spanning the period 1980–2015, divided into 5-y subperiods. The GDP and capital stock data are sourced from the Penn World Table 9.0 (29), while labor force, working-age population, and total population figures are from The World Bank’s World Development Indicators. Human capital is approximated making use of the share of persons attaining at least completed junior secondary, i.e., postprimary education, employing the data from the Wittgenstein Centre for Demography and Global Human Capital. All of the variables which enter the model in levels are measured in the initial year of the corresponding 5-y period, and all specifications include both country and period fixed effects to account for time-invariant characteristics at the country level, as well as for global period shocks.

The first column in Table 1 presents the results of the estimation of a simple regression model where the growth rate of GDP per capita is explained by the growth of capital per worker, the growth of the labor force, and the growth rate of population. In this specification, only the growth rate of physical capital per worker and (marginally) the growth rate of the labor force show significant positive effects on economic growth. Expanding the model to include labor force participation and the working-age share, as well as the initial level of income per capita, we only find additional significant effects on economic growth in the form of conditional income convergence to country-specific equilibria and human capital accumulation effects. The third column of Table 1 presents the estimates obtained after adding the interaction of human capital and initial income to the specification to address potential technology adoption effects, as in Benhabib and Spiegel (14, 15). The results of the estimation of this model unveil significant technology adoption effects fueled by education, with the returns in terms of increased economic growth being larger for poorer economies. Finally, the last column in Table 1 presents the results from the full model, which includes effects of age structure that are determined by the level of human capital of the country. In this model, the role of age structure changes in economic growth depends on whether these happen in the context of high or low human capital stocks.

**Table 1.** Regression results

	(1)	(2)	(3)	(4)
Growth of capital per worker	0.673***	0.559***	0.534***	0.476***
	(8.92)	(5.59)	(5.58)	(5.55)
Growth of labor force	0.436*	0.266	0.262	0.390
	(1.76)	(1.03)	(1.05)	(1.48)
Growth of population	−0.0723	0.167	0.325	0.192
	(−0.11)	(0.31)	(0.60)	(0.34)
Log of Labor force/Working-age population		−0.351	−0.240	−0.265
		(−1.52)	(−1.12)	(−0.89)
Log of Working-age population/Total population		0.0813	−0.259	−1.361**
		(0.27)	(−0.78)	(−2.95)
Postprimary education attainment		0.745*	4.474***	9.560***
		(1.96)	(3.52)	(4.86)
Initial income per capita		−0.506***	−0.380***	−0.270**
		(−5.38)	(-3.36)	(−2.24)
Postprimary education attainment*			−0.351**	−0.710***
Initial income per capita			(−3.06)	(−4.83)
Postprimary education attainment*				0.546
Log of Labor force/Working-age population				(0.92)
Postprimary education attainment*				4.339***
Log of Working-age population/Total population				(3.59)
Observations	835	778	778	778
Countries	167	166	166	166
R^2^ (within)	0.252	0.509	0.527	0.558
Adjusted R^2^ (within)	0.246	0.501	0.519	0.550

Estimates are based on specifications nested in Eq. **1**. All models are based on a panel dataset with 5-y periods, country fixed effects, and period fixed effects included in all models. T-test statistics are based on robust SEs in parentheses; */**/*** stands for significance at the 10%/5%/1% level.

**Fig. 2.**
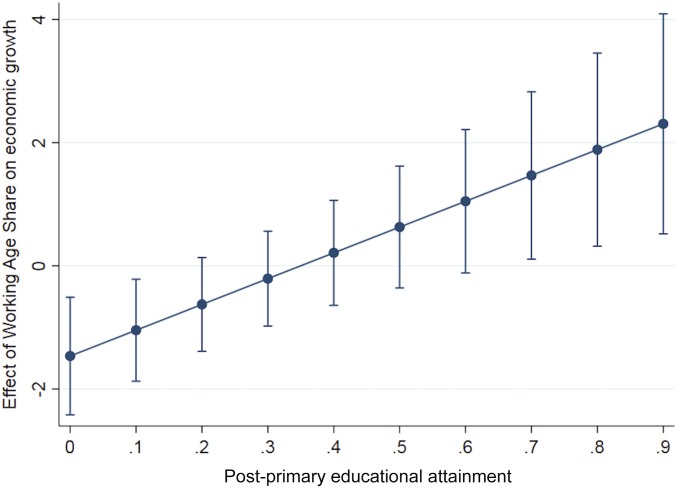
Effect of working age share on GDP per capita growth by level of education.

The effects of changes in the share of the working-age population by level of human capital which are implied by the estimates of this model are depicted in Fig. 2. They indicate that significant positive growth effects of increases in the share of population in working age are only prevalent in countries where a relatively large part of the population has achieved an educational attainment level beyond primary education. The effects of expanding the working-age share that the model predicts for countries with a very low proportion of persons with some secondary education are even negative.

The results obtained for the full sample concerning the role of human capital as a determinant of the effect of age structure on economic growth appear robust to different definitions of the human capital variable, in terms of focusing both on narrower age groups and on female and male education separately. The conclusions also do not change when the model is estimated for different subgroups of countries with a particular focus on those that went through rapid fertility declines during the observation period. The effect of education turns out to be even stronger when the analysis is limited to the early demographic transition countries where changing age structure would only have positive effects on growth for relatively high levels of educational attainment. A detailed discussion of these sensitivity analyses and alterative specifications can be found in *SI Appendix*.

These empirical results confirm and expand the previous findings in Crespo Cuaresma et al. (13) concerning the lack of an independent effect of age structure on productivity and thus on aggregate economic growth. Moreover, explicitly addressing the interactions between the effect of changing age structure and education levels reveals that, in the case of low shares of population having at least completed junior secondary education, the effect can be negative. In other words, a population in which the number of children declines and thus the proportion in working age increases is worse off than in the case of no such change if the education level of the population is low. If the average level of education is relatively high, the results show that also a strong demographic dividend can be reaped from the interaction of more people in working age and those people being better educated.

These findings also suggest that the widely used hierarchical conceptualization of the demographic dividend as an opportunity that is opened and triggered by a decline in the youth dependency ratio and which requires investments in education and health as a second-order additional investment is misleading. The analysis reveals that the age structural change by itself does not open any specific opportunity and the improvement of human capital is the primary and dominant driver of the true demographic dividend.

## The Contribution of Education and Age Structure Changes to Economic Development: The Cases of South Korea and Nigeria

**Fig. 3.**
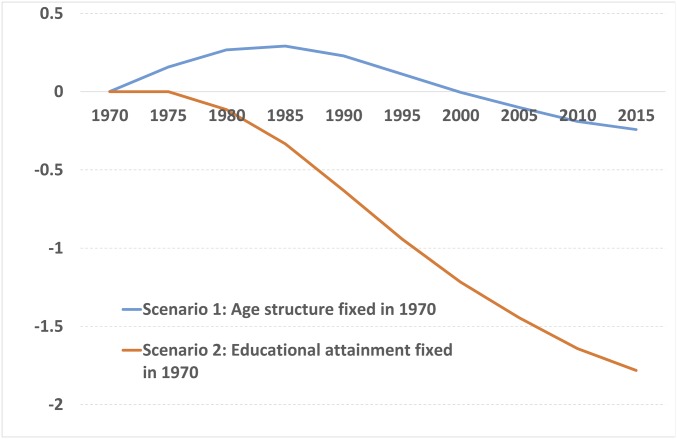
Simulated GDP per capita paths in South Korea by scenario (difference in log-GDP per capita from model fitted values for changing age structure and education).

To assess quantitatively the relative importance of changes in age structure and education as determinants of long-term economic development trends, we employ the estimates of our model to simulate alternative GDP per capita histories for South Korea and Nigeria as two prominent examples of economies at very different stages of their development. In particular, for South Korea, we aim at measuring the contribution of different demographic changes, employing two scenarios. In scenario 1, we fix the share of working-age population in the year 1970 and simulate GDP per capita (using the above model which has been reestimated to include exclusively statistically significant variables) and let all other variables in the model vary as observed in the period 1970–2015. In scenario 2, we obtain GDP per capita estimates by fixing the human capital variable in 1970 and letting the rest of the variables change over the period 1970–2015. Fig. 3 presents the results of these two simulations as log-deviations of the GDP per capita paths implied by the two scenarios compared with that obtained if age structure and education trends are allowed to change as they did in the 45 y considered. The simulation results highlight the quantitative importance of human capital accumulation as a determinant of GDP per capita trends: Without the educational improvement that took place in the country in this period, income per capita in South Korea today would be approximately one-third of its actual value. The income per capita loss implied by fixing age structure in 1970, on the other hand, is extremely small for 2015, and this scenario implies, for some decades, even higher GDP per capita than in the scenario which incorporates the actual dynamics of all variables.

**Fig. 4.**
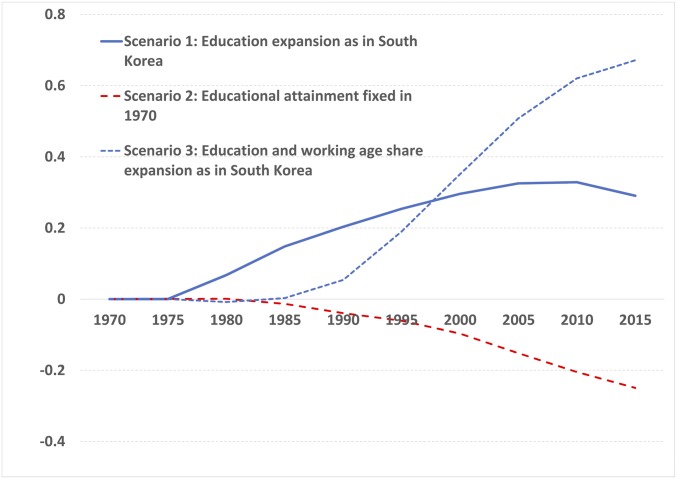
Simulated GDP per capita paths in Nigeria by scenario (difference in log-GDP per capita from model fitted values for changing age structure and education).

We also perform GDP per capita simulations for Nigeria, the most populated country in Africa and an economy whose future development will be central to global poverty dynamics (30). To isolate the differential effects that education and age structure changes may have on economic growth in the country, we calculate counterfactual GDP per capita paths for three different scenarios. In scenario 1, we simulate an expansion of educational attainment in the country similar to the one which took place in South Korea in the period 1970–2015, keeping the age structure dynamics similar to those which took place in Nigeria. Scenario 2 also assumes age structure changes similar to those that actually took place in the country, but fixes the educational attainment level to that in Nigeria in 1970 for the full simulation period. Scenario 3 imposes both the educational attainment and age structure changes that took place in South Korea on Nigeria. Fig. 4 presents the simulation results for these scenarios as deviations from the GDP per capita path implied by the model for the actual developments in the corresponding variables in Nigeria.

The results of the simulations for Nigeria exemplify the importance of human capital as a catalyst of the effect of age structure changes on economic growth. The simulation for a scenario where educational attainment is assumed to expand rapidly following the path experienced by South Korea combined with the actual changes in age structure in Nigeria (scenario 1) results in significantly larger GDP per capita levels by 2000–2015 than given by the actual development. If the Korean education expansion is furthermore combined with the Korean trend in the working-age share (scenario 3), then GDP per capita would first increase less and, after 1995–2000, more than under scenario 1. This is a consequence of the interaction between age structure and education: In a low-education context, an increase in the working-age population leads to a GDP depressing effect while, in a high-education context (after 1995), it leads to an enhancing effect. The simulated GDP per capita in 2015 for Nigeria would be ∼65% higher than the benchmark under scenario 3 and 29% higher under scenario 1. Scenario 2, where educational attainment is fixed at the level it had in Nigeria in 1970 combined with the empirical age structure change, suggests a counterfactual GDP per capita 25% below what has been observed.

These simulation exercises for South Korea and Nigeria demonstrate that improvement in the educational attainment composition of the population is the primary driver and facilitator of economic growth, with age structure changes playing a secondary role. Such counterfactual simulations which independently vary the two different demographic structures have to be interpreted with caution, however, since, in reality, the two changes are not independent. Improvements in female education are widely considered to be a key determinant of declining fertility rates, and hence an application of the South Korean education expansion to Nigeria from 1970 onward would have most likely resulted in a significant fertility decline which also—with some time lag—would have increased the proportion in working age. However, whether the fertility decline leading to a changed age structure is due to exogenous forces (such as family planning programs) or a consequence of improving female education is a separate research question beyond the scope of this paper and does not affect the findings presented here. If, indeed, fertility decline had been induced by external forces and this should have an independent effect on economic growth, then the age structure should have a significant effect independent of the adult education in the same year. A more systematic discussion of the causal mechanism involved and the possible quantity−quality trade-offs in determining the sizes and the education levels of specific cohorts is given in *SI Appendix*.

## Policy Implications

In this paper, we present scientific evidence that puts into question the currently dominant rationale for linking demographic trends with economic growth in developing countries. We show that exogenously induced declines in fertility which result in a higher proportion of the population of typical working age bring, by themselves, no economic growth dividend. Actually, drops in fertility may lead to worsening economic conditions if they happen in the context of very low education, presumably with the increasing proportion of young adults with low education and less family duties having the potential of causing political and economic insecurity. A link can possible be drawn to the extensive literature on the negative trends and security risks associated with the youth bulge (31), although a study of this issue goes beyond the scope of this paper.

Our study confirms earlier analyses showing that improvements in the educational attainment structures of populations are a key driver of economic growth (12, 13, 16). Given that variations in the educational composition of the population can also be denoted demographic changes, one can say that investments in human capital bring the true demographic dividend.

The resulting policy focus on human capital formation is fully in line with the Sustainable Development Goals (SDGs) and, in particular, Goals 3 to 5 on health, education and gender equity. Population growth and age structure are not explicitly mentioned in the SDGs, but reproductive health is listed as one of the more specific targets under the health goal. The findings presented here endorse these global policy priorities. They do not diminish the importance of reproductive health and rights from a human rights perspective, but they imply that attempts to justify them in terms of economic benefits from possibly resulting fertility declines are not substantiated.

The findings do not imply that there should not be any specific population policies. Quite the opposite, they strongly suggest that an explicit policy focus on strengthening societies’ human resources (the number of people by age, gender, education, health status, and labor force participation) should be a development priority. All bigger companies have clear policies for human resource management. Similarly, population policies should focus on national human resource management. Also, given that human capital clearly strengthens societies’ resilience and adaptive capacity to already unavoidable environmental changes (32), this suggests that population policies understood in this way should actually be a priority policy toward sustainable development in rich and poor countries alike.

## Supplementary Material

Supplementary File

## Footnotes

*The IUSSP had, for many years, the following definition of demography prominently on its website: “Demography is the scientific study of changing population size and structures.” It has recently been replaced by the even more general definition, “Demography is the scientific study of human populations,” attributed to its past president Peter McDonald.
