# Supplementary material for: Education rather than age structure brings demographic dividend
Source: Proc Natl Acad Sci U S A. 2019 Jun 10;116(26):12798–803. doi: 10.1073/pnas.1820362116 (PMC6600906; doi:10.1073/pnas.1820362116)
Supplement: Supplementary File [file pnas.1820362116.sapp.pdf]

PNAS
www.pnas.org

Supplementary Information for

Education rather than age-structure brings demographic dividend

Wolfgang Lutz, Jesus Crespo Cuaresma, Endale Kebede, Alexia Prskawetz, Warren C. Sanderson, Erich Striessnig

Wolfgang Lutz

Email: lutz@iiasa.ac.at

This PDF file includes:

Supplementary text and tables

www.pnas.org/cgi/doi/10.1073/pnas.1820362116

The demographic dividend: A theoretical setting

We adopt the theoretical framework put forward by Kelley and Schmidt (2005) and expand it to account for technology adoption effects such as those predicted by the model settings proposed in Benhabib and Spiegel (1994, 2005) or Lutz et al. (2008). The production function of the economy is assumed to be given by a Cobb-Douglas specification with Hicks-neutral technical change,

$$Y_{it} = A_{it} K_{it}^{\alpha} L_{it}^{1-\alpha}, \tag{1}$$

where Y_{it} is total output in country *i* at time t, A_{it} is total factor productivity, K_{it} is the capital stock and L_{it} is total labor input. This implies a specification in growth rates of per-capita variables which is given by

$$\Delta \ln y_{it} = \Delta \ln A_{it} + \alpha \Delta \ln k_{it}. \tag{2}$$

where $y_{it} = Y_{it}/L_{it}$ denotes GDP per worker and $k_{it} = K_{it}/L_{it}$ is capital per worker. GDP per worker can be written as $y_{it} = \frac{Y_{it}}{L_{it}} = \frac{Y_{it}}{N_{it}} \frac{N_{it}}{L_{it}}$, where N_{it} denotes total population, which allows us to rewrite equation (2) in terms of GDP per capita growth instead of GDP per worker growth,

$$\Delta \ln \frac{Y_{it}}{N_{it}} = \Delta \ln y_{it} + \Delta \ln L_{it} - \Delta \ln N_{it} = \Delta \ln A_{it} + \alpha \Delta \ln k_{it} + \Delta \ln L_{it} - \Delta \ln N_{it}. \tag{3}$$

Expanding to the specification used in Crespo Cuaresma et al. (2014), we start by assuming conditional convergence dynamics and a potential direct effect of human capital in the form of education on income growth. This implies that the specification in equation (3) can be expanded to include the lagged level of GDP per worker and a measure of the level of human capital as additional determinants of GDP per capita growth. In addition, following the theoretical mechanisms related to technology adoption described in Benhabib and Spiegel (1994, 2005), we assume that the speed of income convergence, given by the parameter associated to the lagged GDP per worker variable, can be affected by the level of human capital of the country. This leads to a specification where the growth rate of total factor productivity is replaced by its determinants (human capital and the conditional convergence term),

$$\Delta \ln \frac{Y_{it}}{N_{it}} = \theta h_{it} + \mu(h_{it}) \ln y_{it-1} + \alpha \Delta \ln k_{it} + \Delta \ln L_{it} - \Delta \ln N_{it}, \tag{4}$$

where h_{it} is the level of human capital. Further assuming a linear effect of the human capital variable on the speed of convergence, $\mu(h_{it}) = \mu_0 + \mu_1 h_{it}$, and considering the decomposition given by $y_{it-1} = \frac{Y_{it}}{L_{it}} \frac{L_{it}}{W_{it}} \frac{W_{it}}{N_{it}}$, where W_{it} is the population in working age, we get the final specification, which combines the independent effects of age structure and human capital, as well as those caused by their interaction,

$$\Delta \ln \frac{Y_{it}}{N_{it}} = \theta h_{it} + \mu_0 \ln \frac{Y_{it-1}}{N_{it-1}} + \mu_1 h_{it} \ln \frac{Y_{it-1}}{N_{it-1}} - \mu_0 \ln \frac{W_{it-1}}{N_{it-1}} - \mu_1 h_{it} \ln \frac{W_{it-1}}{N_{it-1}} - \mu_0 \ln \frac{L_{it-1}}{W_{it-1}} -$$
$$-\mu_1 h_{it} \ln \frac{L_{it-1}}{W_{it-1}} + \alpha \Delta \ln k_{it} + \Delta \ln L_{it} - \Delta \ln N_{it}. \tag{5}$$

This model predicts effects of age structure which are determined by the prevailing level of human capital. From a theoretical point of view, the parameter μ_1, which measures the effect of human capital on the speed of income convergence, is expected to be negative, reflecting that, *ceteris paribus*, relatively poorer economies benefit more of human capital accumulation than richer ones due to the technology adoption effects, which speeds the process of convergence to the prevalent technology frontier (Benhabib and Spiegel, 1994, 2005). A negative μ_1 implies that the effects of age structure, as captured by the share of working age population on economic growth, depend on the stock of human capital, with higher human capital stocks leading to larger positive effects of increases in working age population relative to total population.

Reconstructing human capital stocks

The main human capital variable of interest in our analysis derives from national level time series on educational attainment by age and sex. As described in the main article, the lack of such information has long been identified as one reason for why stronger effects of human capital on economic growth have not been identified earlier. While time series on school enrollment are readily available for large numbers of countries, comparable time series on education stocks among adult populations with age and sex detail have not been available until recently. Reconstruction efforts using interpolation or the perpetual inventory method often suffer from the lack of quality of the underlying base data, leading to unexplainable jumps in the derived time series and large scale inconsistencies in regression analyses conducted using such datasets (1, 2).

Making use of demographic multi-state cohort component methodology (3, 4), Lutz et al. (5) suggest an alternative approach to reconstructing education stock data. Education carries the methodological advantage of being – for the most part – persistent over the life course. With the exception of unidirectional transition probabilities between different education levels at younger ages, which can be modelled easily from available school enrollment data, no further assumptions are necessary to reconstruct past attainment levels within a cohort. Knowing the proportion of people with a specific level of education aged 50-54 today, we can easily derive the proportion aged 45-49 with the same level of education 5 years earlier.

Thanks to the remarkable degree of inertia in the population system, resulting from life expectancies ranging well beyond 70 years in the majority of countries today, this demographic reconstruction technique can be applied reasonably for decades into the past. Repeated rounds of back-projections with continuously improving baseline data for an increasing number of countries, as well as improved methodology have led to the reconstruction of past population by age, sex, and level of educational attainment back to 1950 in 170 countries (6–8). The resulting database, that has been cross-validated against alternative sources of information where available and for internal consistency (9, 2), is regularly updated and freely available online from the Data Explorer[1] of the Wittgenstein Centre for Demography and Global Human Capital (WiC).

Sensitivity Analysis with Different Education Indicators

Table A1 shows sensitivity analyses in terms of results of the full model as described in the main text applied to different education indicators. While the main model in the main text uses the educational attainment of the total population above age 25 (proportion with post-primary education) the first two columns of the table give the comparable proportions for the age groups 20-39 and 40-64. The results are qualitatively identical to those shown in the main paper in comparison of the two age groups the education effects are somewhat stronger for the middle-aged adults (40-64) than for the young adults (aged 20-39). The third column shows the results if the full model is applied only to the proportion of the population aged 25+ of women and men respectively. Again, the same pattern of results is confirmed. The last column finally gives the results for the mean years of schooling of the total population above age 25. Here the size of the coefficients are not directly comparable to the other columns because of a different metric (years of schooling rather than a proportion) but the results confirm the same story as described for the main model.

[1] http://witt.null2.net/shiny/wic/

Table A1: Regression results by education variable	Age group 20-39	Age group 40-64	Female education	Male education	Mean Years of Schooling
Growth of capital per worker	0.459***	0.467***	0.488***	0.441***	0.474***
	(5.429)	(5.889)	(6.176)	(5.781)	(5.253)
Growth of labour force	0.327	0.360	0.365	0.335	0.236
	(1.245)	(1.466)	(1.460)	(1.291)	(0.886)
Growth of population	0.089	0.136	0.229	0.126	0.235
	(0.155)	(0.243)	(0.413)	(0.215)	(0.417)
Log of Labour force/Working age population	-0.093	-0.091	-0.244	-0.109	-0.058
	(-0.259)	(-0.305)	(-0.827)	(-0.266)	(-0.168)
Log of Working age population/Total population	-0.968	-0.903***	-1.215***	-1.566**	-1.508*
	(-1.578)	(-2.606)	(-3.027)	(-2.549)	(-1.736)
Educational attainment	4.251***	6.135***	9.293***	6.499***	0.393**
	(2.793)	(5.423)	(5.271)	(3.776)	(2.204)
Initial income per capita	-0.291**	-0.312***	-0.304***	-0.212	-0.278*
	(-2.035)	(-2.802)	(-2.788)	(-1.560)	(-1.954)
Educational attainment* Initial income per capita	-0.356***	-0.470***	-0.701***	-0.544***	-0.033***
	(-2.842)	(-5.462)	(-5.341)	(-3.913)	(-2.745)
Educational attainment* Log of Labour force/Working age population	-0.013	0.086	0.487	0.162	-0.018
	(-0.032)	(0.215)	(0.808)	(0.273)	(-0.478)
Educational attainment* Log of Working age population/Total population	2.897***	3.334***	4.018***	4.207***	0.272**
	(3.052)	(4.164)	(3.789)	(3.964)	(2.330)
Observations	778	778	778	778	766
Countries	166	166	166	166	154
Adj. R^2 (within)	0.508	0.535	0.553	0.530	0.510

Estimates based on equation (1). All models based on a panel dataset with 5-year periods, country fixed effects and period fixed effects included in all models. T-test statistics based on robust standard errors in parenthesis. */**/*** stands for significance at the 10%/5%/1% level.

These calculations with different education variables referring to different age-cohort as well as men and women separately also help to resolve some possible concerns about endogeneity. As mentioned in the main text, increasing education may at the same time be reflected in higher human

capital in the main model but also be reflected indirectly through having led to lower fertility and thus to a lower youth dependency ratio. One might also expect that through quantity-quality trade-offs made be parents with respect to choosing the number of children and their education the two aspects might not be independent. When discussing the role of education on fertility at years before time t and the quality-quantity trade-offs in parents' decisions to have fewer but better educated children, it is useful to study the issue by distinguishing between the human capital of the different cohorts involved. In particular, we should distinguish between (a) the quantity-quality relationship within the children's generation and (b) the effect of parents' education (particularly of women) on their fertility, i.e. the size of the next generation.

(a) Within each generation (cohort), the quantity-quality trade-offs decisions made by their parents are reflected in the relationship between cohort size and average education of that cohort. This relationship is an interesting topic in its own right. In the spirit of the Easterlin hypothesis(10), a larger cohort size is a disadvantage and one would expect lower average education. Other theoretical settings would lead to the opposite conclusion and, to our knowledge, the question is still open to some degree - but this question clearly is beyond the scope of this paper. What is relevant for this paper, however, is the fact that possible negative or positive correlations between cohort size and cohort education of any given cohort do not affect our model because they do not both enter it at the same time. The human capital variable that enters the model is the educational attainment of the adult population above age 15. For the younger cohorts (age 0-14 at time t), only their size and not their educational attainment enters the model through the ratio of total population to working age population. Since the mentioned trade-offs can only be within a given generation, the fact that there is little to no overlap between the changing size of the young cohorts (0-14) and the education of the older ones (15+), such possible intra-generation trade-off does not create endogeneity problems in the model.

(b) The second aspect, namely that a higher education of parents affects fertility in the next generation, is a different matter and it is implicit in the model since the parents' education is included in the model through the educational attainment of the 15+ population and their fertility is reflected in the size of the young age group (0-14). – This fact has been stated in the discussion section of the previous draft by saying: "The fact that female education likely influenced fertility some time ago and is thus indirectly reflected in the current age structures only strengthens the case for the important role of education." While this certainly remains true, it may indeed be desirable to shed more light on this in terms of differentiating between the direct effects of education on economic growth and possible indirect ones via age distribution.

    One way of analytically differentiating between the two possible effects of education is to distinguish between male and female education and to include only male education as the human capital variable in the model because it is assumed to matter for economic growth but much less so for fertility. In the literature, it has been repeatedly demonstrated that for fertility female education matters more than male (where the higher income associated with more education may actually produce a positive income effect). To update the evidence on this, we have done some statistical analysis on the link between female and male education and fertility across all recent Demographic and Health Surveys in Sub-Saharan Africa.

    The results are shown in Table A2. They are given for selected countries as well as all of Sub-Saharan Africa together and show the bi-variate relationships between children ever born to

women aged 40-45 (Table A3 for age 30-35) by male and female education as well as after controlling for partners' education. As expected, consistently, female education matters much more than male education. And once female education is controlled for, the effect of male education becomes largely insignificant. Men with primary education in some countries actually show a slightly positive education effect on fertility after controlling for their wives' education (as expected based on a positive income effect). This assessment makes it meaningful to have an alternative estimation of the economic growth model in which the education variable is based on males instead of the total population. Table A1 above shows in the results for the different education variables. A comparison across them shows that the findings are very robust and in particular the findings for male and female education are almost identical.

In any case, the sensitivity analysis described here only refers to the case that fertility decline had been predominantly induced by education of mid-aged adults. If fertility decline had been induced by external forces such family planning programs – as has been the reasoning of UNFPA and others pointing at the demographic dividend mechanism – then a falling youth dependency should have a significant effect in the model independent of the education of middle-aged adults. But the empirical results do not support such an independent effect.

Table A2: Regression results of the impact of male education vs female education on the cumulative number of children born by women **aged 40-44** for selected sub-Saharan Africa countries as well as the aggregate results for all SS-African countries with a DHS. Data are obtained from the most recent demographic and health surveys of each country

	Nigeria				***Ethiopia***			
Variables	**Bi-Variate**		**Adjusted**		**Bi-Variate**		**Adjusted**	
	**IRR**	**95% CI**	**IRR**	**95% CI**	**IRR**	**95% CI**	**IRR**	**95% CI**
**Partner Education**								
No Education	1.00		1.00		1.00		1.00	
primary	0.93	0.90-0.95	0.96	0.94-0.99	0.96	0.93-0.99	1.00	0.97-1.02
Some Secondary or more	0.71	0.79-0.83	0.92	0.89-0.95	0.61	0.58-0.63	0.78	0.74-0.84
**Women Education(ref=no)**								
No Education	1.00		1.00		1.00		1.00	
primary	0.95	0.93-0.98	0.98	0.96-1.01	0.81	0.78-0.84	0.86	0.82-0.89
Some Secondary or more	0.72	0.71-0.74	0.76	0.74-0.79	0.50	0.47-0.53	0.61	0.57-0.59

	Kenya				***Niger***			
Variables	**Bi-Variate**		**Adjusted**		**Bi-Variate**		**Adjusted**	
	**IRR**	**95% CI**	**IRR**	**95% CI**	**IRR**	**95% CI**	**IRR**	**95% CI**
**Partner Education**								
No Education	1.00		1.00		1.00		1.00	
primary	0.95	0.92-0.98	1.00	0.97-1.04	1.00	0.94-1.06	1.03	0.97-1.09
Some Secondary or more	0.70	0.68-0.73	0.85	0.81-0.89	0.78	0.73-0.83	0.87	0.81-0.93
**Women Education(ref=no)**								
No Education	1.00		1.00		1.00		1.00	
primary	0.89	0.86-0.91	0.91	0.89-0.94	0.92	0.87-0.97	0.94	0.89-0.99
Some Secondary or more	0.61	0.58-0.63	0.68	0.65-0.71	0.70	0.65-0.76	0.76	0.69-0.83

	Ghana				***All sub-Sahara Africa countries***			
Variables	**Bi-Variate**		**Adjusted**		**Bi-Variate**		**Adjusted**	
	**IRR**	**95% CI**	**IRR**	**95% CI**	**IRR**	**95% CI**	**IRR**	**95% CI**
**Partner Education**								
No Education	1.00		1.00		1.00		1.00	

primary	0.95	0.91-1.01	0.97	0.93-1.02	0.97	0.96-0.97	1.00	0.99-1.00
Some Secondary or more	0.73	0.71-0.76	0.84	0.80-0.87	0.77	0.77-0.78	0.89	0.88-0.90
**Women Education(ref=no)**								
No Education	1.00		1.00		1.00		1.00	
primary	0.92	0.89-0.96	0.97	0.93-1.01	0.93	0.93-0.94	0.95	0.95-0.96
Some Secondary or more	0.67	0.65-0.70	0.76	0.72-0.79	0.68	0.68-0.69	0.74	0.73-0.74

Results are obtained from community (and country in the case of all SSA countries) fixed effect poison regression model estimates

Table A3: Regression results of the impact of male education vs female education on the cumulative number of children born by women **aged 30-34** for selected sub-Saharan Africa contraries as well as the aggregate results for all SS-African countries with a DHS. Data are obtained from the most recent demographic and health surveys of each country

Variables	***Nigeria***				***Ethiopia***			
	**Bi-Variate**		**Adjusted**		**Bi-Variate**		**Adjusted**	
	**IRR**	**95% CI**	**IRR**	**95% CI**	**IRR**	**95% CI**	**IRR**	**95% CI**
**Partner Education**								
No Education	1.00		1.00		1.00		1.00	
primary	0.92	0.90-0.95	1.01	0.98-1.03	0.94	0.92-0.97	0.99	0.97-1.02
Some Secondary or more	0.74	0.72-0.75	0.91	0.88-0.93	0.60	0.58-0.62	0.80	0.77-0.84
**Women Education(ref=no)**								
No Education	1.00		1.00		1.00		1.00	
primary	0.89	0.87-0.91	0.91	0.89-0.93	0.82	0.80-0.85	0.86	0.83-0.89
Some Secondary or more	0.64	0.62-0.65	0.68	0.66-0.70	0.48	0.45-0.50	0.56	0.53-0.59

Variables	***Kenya***				***Niger***			
	**Bi-Variate**		**Adjusted**		**Bi-Variate**		**Adjusted**	
	**IRR**	**95% CI**	**IRR**	**95% CI**	**IRR**	**95% CI**	**IRR**	**95% CI**
**Partner Education**								
No Education	1.00		1.00		1.00		1.00	
primary	0.88	0.85-0.91	0.97	0.93-1.01	0.92	0.88-0.97	0.96	0.91-1.00
Some Secondary or more	0.66	0.64-0.69	0.83	0.79-0.87	0.68	0.65-0.71	0.80	0.75-0.84
**Women Education(ref=no)**								
No Education	1.00		1.00		1.00		1.00	
primary	0.83	0.81-0.86	0.87	0.84-0.90	0.87	0.84-0.91	0.92	0.88-0.96
Some Secondary or more	0.58	0.56-0.60	0.65	0.62-0.68	0.62	0.58-0.66	0.72	0.67-0.77

Variables	***Ghana***				***All sub-Sahara Africa countries***			
	**Bi-Variate**		**Adjusted**		**Bi-Variate**		**Adjusted**	
	**IRR**	**95% CI**	**IRR**	**95% CI**	**IRR**	**95% CI**	**IRR**	**95% CI**
**Partner Education**								
No Education	1.00		1.00		1.00		1.00	
primary	0.97	0.93-1.01	1.01	0.96-1.05	0.94	0.94-0.95	0.98	0.97-0.99
Some Secondary or more	0.72	0.70-0.75	0.86	0.82-0.90	0.73	0.73-0.74	0.86	0.86-0.87
**Women Education(ref=no)**								
No Education	1.00		1.00		1.00		1.00	
primary	0.92	0.88-0.95	0.95	0.91-0.99	0.90	0.90-0.91	0.93	0.93-0.94
Some Secondary or more	0.65	0.62-0.67	0.72	0.69-0.76	0.63	0.63-0.64	0.69	0.69-0.70

Results are obtained from community (and country in the case of all SSA countries) fixed effect poison regression model estimates

Sensitivity Analysis with respect to different country groupings

While the results as presented in the main text have been obtained for the time series of all 166 countries for which data have been available, one might also ask what specific patterns appear for sub-groups of countries that are at different stages of their demographic transitions. Since the demographic dividend argument applies in particular to countries experiencing rapid fertility declines (for whatever reason) we will focus on countries that went through demographic transition during the observation period 1980-2015. Since there is no standard definition of the level of fertility at which the demographic transition begins and at the level at which it ends nor what is a rapid and what is a fast fertility decline, we applied two alternative definitions that focus on the magnitude and on the speed of decline in TFRs between 1975-80 and 2010-15 according to UN data: Group 1 includes all countries that experienced a decline in TFR of 2.0 or more over this period and Group 2 includes those whose TFR declined by more than 50 percent over the observation period. Group 1 includes 75 countries and Group 2 47 countries. To also cover countries that are still in the earlier phases of demographic transition, we also defined a Group 3 that includes all countries defined by the World Bank as low income countries over the observation period and not having yet experienced the strong fertility declines of either Group 1 or 2. This third group of early transition countries includes 52 countries. The countries belonging to each of the three groups are listed in tables A6 and A7.

The results show for the countries that experienced significant fertility decline over the observation period (Groups 1 and 2) highly significant positive effects of education on economic growth but no significant effects related to age structural changes (see Table A4). For Group 3 of poor early demographic transition countries the education variable is also highly significant (even more than for all countries) and the direct age structure effect is insignificant, while the interaction terms between education and age-structure variables are significant again. These significant interactions indicate that for these countries a relative expansion of the working age population (as expected after a fertility decline) and subsequently of participation rates would only have positive effects on growth for relatively high levels of educational attainment.

In sum, these sensitivity runs for groups of countries that have recently gone through rapid fertility declines and those that are still in early stages reconfirm the general finding that we obtained for the full sample of 166 countries. The dominating role of education in driving economic growth comes out even more strongly for the countries that went through demographic transition over the observation period while none of the age structure variables (not even the interaction with education) appear significant for these countries for which the demographic dividend previously had been considered most relevant.

Table A4: Regression results by stage of the demographic transition	Group 1<br>Countries with a TFR decline of at least 2.0	Group 2<br>Countries with 50% decline in TFR	Group 3<br>Low income countries with early demographic transition
Growth of capital per worker	0.530***	0.498***	0.447***
	(5.370)	(6.413)	(4.937)
Growth of labour force	0.084	0.333	0.496
	(0.270)	(1.161)	(0.928)

Growth of population	0.479	-0.282	2.817**
	(0.831)	(-0.568)	(2.254)
Log of Labour force/Working age population	0.202	-0.128	-0.135
	(0.472)	(-0.238)	(-0.454)
Log of Working age population/Total population	-0.381	-0.206	-1.009
	(-0.633)	(-0.306)	(-1.106)
Educational attainment	10.60***	7.766*	14.45***
	(3.004)	(1.760)	(4.859)
Initial income per capita	-0.379**	-0.170*	-0.115
	(-2.445)	(-1.817)	(-0.713)
Educational attainment* Initial income per capita	-1.053***	-0.666*	-1.046***
	(-3.766)	(-1.752)	(-4.380)
Educational attainment* Log of Labour force/Working age population	-0.751	0.971	2.245**
	(-0.909)	(1.057)	(2.106)
Educational attainment* Log of Working age population/Total population	3.245	-0.174	7.393***
	(1.360)	(-0.070)	(4.502)
Observations	347	215	240
Countries	75	47	52
Adj. R^2 (within)	0.466	0.476	0.715

Estimates based on equation (1). All models based on a panel dataset with 5-year periods, country fixed effects and period fixed effects included in all models. T-test statistics based on robust standard errors in parenthesis. */**/*** stands for significance at the 10%/5%/1% level.

Table A5: Summary statistics for variables used in regression for all 166 countries	Mean	Std. Dev.	Median	Minimum	Maximum	#Obs
Growth of GDP per capita	0.09	0.19	0.09	-1.26	1.26	778
Growth of capital per worker	0.09	0.16	0.08	-0.40	1.85	778
Growth of labour force	0.10	0.08	0.10	-0.21	0.90	778
Growth of population	0.09	0.07	0.09	-0.27	0.86	778
Log of Labour force/Working age population	-0.38	0.16	-0.36	-1.04	-0.04	778
Log of Working age population/Total population	-0.54	0.11	-0.56	-0.79	-0.15	778
Post-primary education attainment	0.33	0.24	0.29	0.00	0.86	778
Initial income per capita	8.78	1.24	8.83	5.09	12.38	778

Table A6: List of countries classified as low income by the World Bank

Low income
Benin
Burkina Faso
Burundi
Central African Republic
Chad
Comoros
D.R. of the Congo
Ethiopia
Gambia
Guinea
Guinea-Bissau
Haiti
Liberia
Madagascar
Malawi
Mali
Mozambique
Nepal
Niger
Rwanda
Sierra Leone
Togo
U.R. of Tanzania: Mainland
Uganda

Table A7: List of countries by fertility changes over the period 1975/80-2010/15

**TFR declined by more than 2.0**		**TFR declined by more than half**	
Comoros	Myanmar	Djibouti	Mexico
Djibouti	Philippines	Mauritius	Nicaragua
Eritrea	Thailand	Rwanda	Brazil
Ethiopia	Viet Nam	Algeria	Colombia
Kenya	Bahrain	Libya	Paraguay
Madagascar	Iraq	Morocco	Peru
Malawi	Jordan	Tunisia	French Polynesia
Mayotte	Kuwait	Western Sahara	
Rwanda	Lebanon	Botswana	
Zambia	Oman	Swaziland	
Zimbabwe	Qatar	Cabo Verde	
Algeria	Saudi Arabia	China, Taiwan Province of China	
Egypt	State of Palestine	Mongolia	
Libya	Syrian Arab Republic	Republic of Korea	
Morocco	Turkey	Uzbekistan	
Sudan	United Arab Emirates	Bangladesh	
Tunisia	Yemen	Bhutan	
Western Sahara	Albania	India	
Botswana	Dominican Republic	Iran (Islamic Republic of)	
Lesotho	Grenada	Maldives	
Namibia	Haiti	Nepal	
South Africa	Saint Lucia	Brunei Darussalam	
Swaziland	Saint Vincent and the Grenadines	Cambodia	
Cabo Verde	Belize	Lao People's Democratic Republic	
Cote d'Ivoire	El Salvador	Myanmar	
Ghana	Guatemala	Thailand	
Liberia	Honduras	Viet Nam	
Senegal	Mexico	Bahrain	
Togo	Nicaragua	Jordan	
Mongolia	Bolivia (Plurinational State of)	Kuwait	
Tajikistan	Brazil	Lebanon	
Turkmenistan	Colombia	Oman	
Uzbekistan	Ecuador	Qatar	

Afghanistan

Bangladesh

Bhutan

India

Iran (Islamic Republic of)

Maldives

Nepal

Pakistan

Brunei Darussalam

Cambodia

Indonesia

Lao People's Democratic Republic

Malaysia

Paraguay

Peru

Venezuela (Bolivarian Republic of)

Papua New Guinea

Solomon Islands

Vanuatu

Micronesia (Fed. States of)

French Polynesia

Samoa

Saudi Arabia

Syrian Arab Republic

Turkey

United Arab Emirates

Albania

Saint Lucia

Saint Vincent and the Grenadines

Belize

Costa Rica

El Salvador

Guatemala

Honduras

References

1. Cohen D, Soto M (2007) Growth and human capital: Good data, good results. *J Econ Growth* 12(1):51–76.

2. Goujon A, et al. (2016) A harmonized dataset on global educational attainment between 1970 and 2060 - An analytical window into recent trends and future prospects in human capital development. *J Demogr Econ* 82(3):315–363.

3. Rogers A (1975) *Introduction to Multiregional Mathematical Demography* (John Wiley & Sons, New York, USA) Available at: http://www.colorado.edu/ibs/pubs/pop/pop2007-0002.pdf.

4. Keyfitz N (1985) *Applied mathematical demography* (Springer Verlag, New York, USA). Second Edition Available at: http://www.springer.com/social+sciences/population+studies/book/978-0-387-22537-1.

5. Lutz W, Goujon A, KC S, Sanderson WC (2007) Reconstruction of populations by age, sex and level of educational attainment for 120 countries for 1970-2000. *Vienna Yearb Popul Res* 2007:193–235.

6. KC S, Barakat B, Goujon A, Skirbekk V, Lutz W (2010) Projection of populations by level of educational attainment, age, and sex for 120 countries for 2005-2050. *Demogr Res* 22:383–472.

7. Lutz W, Butz WP, KC S eds. (2014) *World Population and Human Capital in the Twenty-First Century* (Oxford University Press, Oxford, UK) Available at: http://ukcatalogue.oup.com/product/9780198703167.do.

8. Lutz W, Goujon AV, KC S, Stonawski M, Stilianakis N (2018) *Demographic and human capital scenarios for the 21st century: 2018 assessment for 201 countries* (Publications Office of the European Union, Luxembourg) doi:10.2760/41776.

9. Speringer M, et al. (2015) *Validation of the Wittgenstein Centre Back-projections for Populations by Age, Sex, and Level of Education from 1970 to 2010* (International Institute for Applied Systems Analysis (IIASA), Laxenburg, Austria).

10. Easterlin RAA (1987) *Birth and Fortune: The Impact of Numbers on Personal Welfare* (University of Chicago Press, Chicago). Revised.
